# AI and Its Shifting Roles in the Therapeutic Relationship: Implications for Precision Medicine

**DOI:** 10.3390/jpm16060324

**Published:** 2026-06-17

**Authors:** Michael Igoumenidis, Venetia-Sofia Velonaki

**Affiliations:** 1 Department of Nursing, University of Patras, 26334 Patras, Greece; 2 Department of Nursing, National and Kapodistrian University of Athens, 11527 Athens, Greece; venvel@nurs.uoa.gr

**Keywords:** accountability, artificial intelligence, explainability, replaceability, role transformation, therapeutic relationship

## Abstract

The emergence and increasing use of artificial intelligence (AI) in healthcare have paved the way for highly personalized and time-saving approaches in the field of precision medicine. It can be applied to determine a prognosis, diagnosis, and recommended treatment, and may also be used for patient monitoring. As AI applications become more widely available, reliable and easy to use, they are rapidly reshaping the traditional roles of professionals and patients in the therapeutic relationship. On the positive side, professionals may have more time to communicate with patients and provide individualized care, whereas patients may become more empowered and autonomous due to AI-facilitated personalized information and monitoring. On the negative side, AI applications threaten to reduce the role of professionals to a mediating one in clinical decision-making, provide patients with misinformation, and lead to misunderstandings that hinder patients’ autonomy. In this narrative review, we examine the main ethical issues related to the AI-induced shift in roles in the therapeutic relationship, within four inter-related themes: the validity of claims that algorithms outperform humans in certain tasks; the ways in which AI saves time for health professionals but also takes time to properly explain and implement; the issues of trust and accountability, especially if AI suggestions lead to patient harm; and what AI’s alleged cost-effectiveness means for professionals’ employment and remuneration. Across the three roles, we find a common pattern: AI tends to absorb the technical and data-processing parts of clinical work while leaving its relational core to humans. Physicians move toward oversight and interpretation, nurses retain the attentiveness and responsiveness that define care, and patients gain tools for self-management that can widen autonomy or, left unguided, erode it. Whether the overall effect is benign depends less on the technology than on how outperformance is evidenced, how the freed time is used, how trust and accountability are anchored in people, and how cost pressures are managed. The article concludes with some suggestions for prudent use of AI in healthcare, indicating the appropriate measures that can be used to harness the power of AI without damaging the traditional cornerstones of the therapeutic relationship.

## 1. Introduction

The emergence of artificial intelligence (AI) in healthcare and the increasing availability of AI-powered tools and services in various clinical settings have the potential to drastically change conventional methods of delivering efficient and patient-centered healthcare, with improved outcomes for patients, practitioners and the system as a whole [1]. Simply put, AI consists of algorithms which machines use to mimic and arguably surpass human intelligence in terms of learning, adapting and problem-solving, thanks to their ability to synthesize big amounts of data from multiple sources in a fast and reliable manner [2]. In healthcare, these algorithms can have significant contributions in three broad categories: clinical decision-making, patient monitoring and hospital organizational management [3].

Three terms recur throughout this article and need to be fixed at the outset. We use the term precision medicine to refer to the tailoring of prevention, diagnosis and treatment to the molecular, genetic and clinical profile of each patient. Personalized care is the wider practice of adapting clinical decisions and communication to a patient’s preferences, values and circumstances, whereas individualized care refers to the concrete delivery of that adapted plan in everyday clinical contact. The terms are related but not interchangeable: precision medicine supplies the data-driven inputs, while personalized and individualized care concern how those inputs are translated into the encounter between professional and patient. That encounter is what we call the therapeutic relationship, understood here as the purposeful, trust-based connection between a health professional and a patient, built through communication, empathy and responsiveness, which serves as the foundation of patient-centered care [4].

Clinical decision-making can be improved by AI’s capability to accurately predict and diagnose diseases or complications, processing and interpreting large datasets which human practitioners cannot manage without devoting excessive time and energy to the task. For example, AI can be used in cardiovascular imaging AI to complete laborious tasks such as image segmentation, analysis, and integration with other clinical data, thus improving patient selection, diagnosis and risk stratification [5]. In addition, AI can help with the design of tailored treatment and care plans based on each individual patient’s unique genetic and metabolic profile, and can even enable personalized drug design in a fast, efficient, and relatively cheap way [6]. To this end, the study of pharmacogenomics is of central importance since it predicts the involvement of gene interactions and other personalized data in medication effects and side effects, thus allowing “precision prescribing”—that is, therapies tailored to specific needs of individual patients, rather than treatment plans based on drugs and dosages that apply to groups of patients [6]. For instance, AI can specify the correct oncology treatment method based on the identification of particular biological molecules (biomarkers) of tumor cells, thus enhancing prognosis and response prediction at an individual level [7].

Inter-related to clinical decision-making, patient monitoring can also benefit from AI in many ways. AI-driven predictive analytics are used in patient monitoring systems, such as wearable devices and periodic health examinations; combined with a constant flow of the latest medical evidence and recommendations, this intense monitoring can result in real-time modifications of treatment plans, which can be tailored according to each individual patient’s progress. This is particularly important for the management of chronic diseases, the dynamic nature of which can cause rapid deterioration of the patient’s status that can be related to environmental factors or lifestyle preferences, as well as the emergence of various expected or unexpected complications. For example, constant monitoring of a diabetic patient enhanced by AI technologies can lead to the real-time prediction of future glucose levels and early detection of adverse glycemic events, and subsequent immediate modifications to the patient’s drug treatment plan and diet [8]. Remote patient monitoring can also detect various patient activities, such as falls and mobility-related illnesses, as well as their adherence to prescribed medications, thus allowing both healthcare practitioners and patient informal carers to rapidly respond to any irregularities, improving outcomes and reducing overall costs [9].

Reduction in healthcare costs and overall efficiency in terms of resource utilization can also be achieved with the implementation of AI in hospital organizational management. Generative AI tools can draft healthcare reports, organize patient data, schedule appointments and procedures, optimize patient flow, and, in general, handle time-consuming bureaucratic duties that create frustration amongst healthcare practitioners [10]. The automation of administrative tasks with AI means that hospital operations can be more efficient, significantly freeing up time for patient care instead of bureaucratic procedures, which in turn leads to reduced waiting times and increased satisfaction among patients [3]. Moreover, AI tools are becoming an increasingly common avenue for automated communication with clients, as AI-powered chatbots and virtual assistants can answer simple questions and send messages for upcoming appointments or medication schedules. Interestingly, researchers point out that, in the future, chatbots will be able to communicate with patients in ways that outperform humans in terms of empathy [11]. Finally, AI-based tools help mitigate waste and abuse in healthcare systems by identifying errors in medical records or incorrect insurance claims, thereby saving resources so that they can be more efficiently used [12]. The promise of cost containment is, in fact, the principal force behind much of this deployment. Health systems facing chronic staff shortages and lengthening waiting lists turn to AI to do more with fewer people, and triage is one of the clearest cases. Machine learning and natural language processing are now used to sort patients by urgency at the point of entry, and prospective studies report gains in triage accuracy and patient flow [13]. Whether such tools genuinely lower total expenditure is contested. Reviews that account for infrastructure, integration and maintenance find that the reported savings are often overstated [14]. We return to this tension below, because the expectation of cheaper care shapes how readily the roles in the therapeutic relationship are allowed to change.

The above-described developments, among others, have already inflicted profound changes on the landscape of healthcare, and they have the potential to exercise even greater influence in the near future, as AI applications become more available, sophisticated, reliable and easy to use. Arguably, the main direction of all these technological advancements is toward increased individualization of healthcare, in both direct and indirect ways. The direct way in which this individualization takes place has to do with the usage of massive amounts of data that AI is capable of: biometric information, genetic data, medical images and laboratory tests, disease patterns, demographics and personal lifestyle choices can all be used to inform clinical decision-making and allow accurate and easily adaptable tailor-made approaches, in patient identification, treatment and monitoring plans. In an indirect way, by handling bureaucratic duties, AI reduces administrative burdens for healthcare practitioners, allowing them to spend more time with patients in contact and employ individualized approaches [10]. Therefore, it can be argued that AI democratizes access to precision medicine and personalized care by making tailor-made plans easily available, feasible and low-cost [2]. These gains, however, represent only one side of the ledger. The same capabilities that redistribute clinical work also raise ethical questions, and it is those questions that decide whether the redistribution helps patients and professionals or harms them.

On the other hand, it cannot be denied that the implementation of AI tools in healthcare entails some serious ethical considerations that need to be addressed. As with any recent technological revolution, the discipline of bioethics has highlighted the main ethical issues with the use of AI in clinical settings, and thus caution and careful reflection are warranted before making AI an indispensable partner. These inter-related ethical issues fall into one of these broad categories [15]:

- Effectiveness, reliability and evaluation: AI does not require strict evaluation, and claims about its effectiveness can be overstated [16].
- Justice, inequality, bias and discrimination: AI algorithms rely on training data that do not sufficiently represent minority groups, thus leading to disparities when it comes to patient identification and subsequent allocation of resources [10].
- Privacy and confidentiality: predictive analytics techniques require a large amount of data, which needs to be adequately protected against potential security breaches [17].
- Machine paternalism and respect for autonomy: AI could reintroduce paternalism if it relies on the values of those who program it, instead of the patients’ values [15].
- Trust and responsibility: to trust AI essentially means to trust the people who designed it, together with those who use it at an organizational and clinical level; yet who is responsible if harm occurs is a complex question that has not been adequately answered [18].
- Need for explanation, justification and transparency: patients should be informed about the use of AI in their treatment plans and provide their consent; however, explaining the rationale behind AI recommendations is often a difficult task, since healthcare practitioners themselves do not fully understand the way that algorithms work (especially “black box” machine learning algorithms) [19].
- Obsolescence, dehumanization and deskilling: there is the fear that AI can render human clinical interactions obsolete; though this fear may be exaggerated, it is undeniable that there will be major transformations in the ways in which healthcare practitioners and patients function within the therapeutic relationship [10].

In this article, we focus on this last category of ethical considerations; namely, how AI reshapes the traditional roles of professionals and patients and what these changes could mean for the future of healthcare. We also take into account other inter-related ethical concerns, such as the issues of trust, responsibility and accountability, which are of central importance in the therapeutic relationship, as well as the issue of machine paternalism. In general, the whole discussion revolves around the question of whether the AI advances that foster the practice of precision medicine and personalized care pose risks for the autonomy of patients and healthcare professionals, and whether their roles could deteriorate due to overdependence on AI applications. We examine existing and prospective changes in the roles of physicians, nurses and patients in separate sections, and we explain the ways in which these modified roles interact in Section 4. We then conclude with some suggestions for the prudent use of AI in healthcare based on the recent literature, indicating the appropriate measures that will hopefully enable the healthcare sector to harness the power of AI without damaging the traditional cornerstones of the therapeutic relationship.

Against this background, the article addresses three central questions. First, how does AI redistribute the traditional roles of physicians, nurses and patients within the therapeutic relationship? Second, how do four factors—the credibility of outperformance claims, the management of clinical time, the conditions of trust and accountability, and the economic pressures of AI—shape whether these shifts strengthen or weaken professional and patient autonomy? Third, what practical and regulatory measures can keep the transformation aligned with the core values of the therapeutic relationship? Earlier reviews have mapped the ethics of AI in healthcare at a broad level [20], yet the narrower question of how shifting roles reshape the therapeutic relationship has received less sustained attention. That is the gap this article addresses. We address the three questions in turn through the role analysis that follows, link them together with the cross-cutting factors in the Discussion, and answer them in the Conclusions.

## 2. Materials and Methods

This article is a narrative review. We chose a narrative approach because the aim is to interpret and synthesize a fast-moving and cross-disciplinary body of work that spans clinical, ethical and policy sources, rather than to estimate a pooled effect from comparable studies. We searched PubMed, Scopus and Google Scholar for studies published mainly between 2019 and 2026, restricted to English, using combinations of the terms artificial intelligence, machine learning, precision medicine, personalized care, therapeutic relationship, doctor–patient relationship, nursing, patient autonomy, trust, accountability, explainability and cost-effectiveness. Priority was given to peer-reviewed studies, reviews and ethics scholarship of direct relevance to the transformation of professional and patient roles, supplemented by professional statements and policy documents where these clarified current debates. Sources were selected for their relevance to the four themes that organize the Discussion rather than through a formal screening protocol, which is consistent with the interpretive aim of a narrative review. Because the review is narrative rather than systematic, we do not report a screening count or a formal quality-appraisal score; we address the resulting trade-off, breadth and interpretive depth set against the reproducibility of a systematic search in Section 5.

## 3. AI and Role Transformation

Essentially, the magnitude of every technological achievement is proportional to the change that it inflicts on people involved. People directly involved in the therapeutic relationship are healthcare professionals on one hand and patients and their relatives on the other. First, we will analyze the impact that AI has on the physician’s traditional role, considering specific tasks and specialties, and we will then consider AI’s influence on nursing. Many of the role transformations that we examine apply in similar ways to physicians, nurses, and all other healthcare professionals. Next, we will present the ways in which AI transforms the patient’s role, also referring to their supportive environment (friends and relatives) in cases where this environment is also involved in the therapeutic relationship.

Table 1 summarizes the direction of these shifts, the main opportunities and risks attached to each role, and the human element that should be protected. The subsections that follow develop each row in turn.

### 3.1. The Changes in the Traditional Role of Physicians

Arguably, the most important change that AI can bring to the practice of medicine is the critical enhancement of the capacity for personalized approaches in prognosis, diagnosis and treatment.

In the field of prognosis, the physician’s role can be significantly reduced to a mediating one. Algorithms using predictive analytics can foretell gene expression for various diseases [21] by working with large datasets, identifying patterns and making prognoses [6]. A physician would not be involved in this process, since AI can use data stored online to automatically identify people at risk, without requiring physicians to be directly involved in screening procedures. They would be simply notified of the results, and undertake responsibility to find the people identified, inform them and discuss options that could reduce the risk that AI estimated. In some other cases of enhanced prediction, physicians may be more actively involved; for instance, AI could be used to predict heart failure hospitalization based on data from wearable devices that physicians have prescribed for specific patients they have examined [22]. However, this does not change the fact that physicians base their decisions on data processing which they are unable to do themselves without devoting excessive time and energy, and that they thus place their trust in predictive analytics rather than in their own data examining and interpretation competencies.

Similar changes can be observed in the field of diagnostics, especially in areas such as dermatology, ophthalmology and radiology—collectively referred to as “vision-oriented specialties” [10]. Advanced machine learning and deep learning algorithms analyze and compare medical images from large datasets, thus identifying abnormalities and pathologies with great accuracy and sensitivity; for instance, detecting melanoma [23], diabetic retinopathy and glaucoma [24], and various types of tumors [25] at very early stages. Since they cannot process the amount of data required for this level of precision, diagnosing physicians’ role may end up consisting of simply validating the algorithm’s results. Some physicians may indeed feel threatened by these developments and be reluctant to follow the recommendations suggested by AI-aided diagnostic systems, but generally this leads to decreased diagnostic performance [26,27].

Apart from diagnostic uncertainty, AI has the potential to also reduce therapeutic uncertainty and enhance personalized treatment approaches. AI-informed therapeutic plans can be highly individualized, shifting the emphasis from standardized clinical protocols that are addressed to groups of patients to data-driven personalized plans. Data refers to genomic considerations (genotype-guided treatment) which could even lead to the discovery of personalized drugs for some cases, clinical considerations, where age, co-morbidities and organ function predicate treatment decisions, as well as social determinants of health for each patient [12]. All these contribute to the achievement of precision prescribing, which reassures patients that they receive therapies adjusted to their specific needs. Furthermore, AI enables fast, real-time modifications in treatment schemes, as various monitoring devices can detect deteriorations or complications, integrating physiological data with electronic health records information [28]. Therefore, the treating physician’s role can be greatly facilitated by AI, but they are at risk of becoming complementary to the algorithmic capabilities of drafting personalized treatment plans.

All of the above-described ongoing changes in the traditional role of physicians come with several critical concerns, the most important being the question of replaceability. Can physicians of the future be entirely replaced by AI systems which will perform their duties in a more precise and efficient manner? This possibility seems far-fetched, as AI currently lacks the capacity to address all that is involved in medical practice. For instance, it is a valuable tool that has recently found its way into the operating theaters, but in the end, it is the surgical team who have control over the whole procedure [29]. In addition, the practice of medicine is deeply intertwined with the therapeutic relationship that is uniquely built, in which the AI’s role cannot be anything more than the physician’s assistant and technical advisor; therefore, it has been claimed that the possibility of replacement of physicians by AI is a pseudo-problem [30]. Still, this certainty is not shared by everyone. For instance, European health leaders have recently warned that “AI tools must be designed and implemented alongside doctors and other healthcare professionals rather than being imposed on them or used to replace them” and called for appropriate regulation [31]. Indeed, it is plausible to assume that the future of the physician’s role is subject to the ways that the scientific community will allow AI to be used and expanded. Cost is rarely absent from this debate. Much of the political enthusiasm for clinical AI rests on the expectation that it will curb spending, whether by raising throughput, shifting tasks away from expensive specialists, or reducing the headcount a service needs. The economic case is real but unsettled. Some analyses project substantial savings in diagnosis and treatment, while systematic appraisals caution that many estimates leave out the cost of infrastructure, integration and training and may therefore overstate the net benefit [14]. For the physician, the relevant point is that decisions about how far to delegate a task to AI are seldom made on clinical grounds alone. They are also budgetary and political, which is why the redistribution of the physician’s role cannot be read as a purely technical matter.

### 3.2. The Changes in the Traditional Role of Nurses

When it comes to the nursing profession, AI is mainly considered as a collaborative tool that enables nurses to engage more deeply in patient care. The nursing shortage is a worldwide problem that has not been adequately addressed, with severe implications for patient safety and care optimization [32]. AI systems can reduce the need for nursing personnel by automating various routine tasks, such as monitoring patient vital signs, record keeping, medication scheduling, and discharge planning [33]. For instance, the Rothman Index is a real-time, EHR-integrated, automated, and AI-driven score that measures a hospitalized patient’s overall clinical acuity and risk, including 11 nursing assessment metrics that are derived from routine nursing documentation [34]. The use of this Index can help nurses have significant impacts on patient care quality, even in settings where resources are limited. Virtual nursing assistants, powered by AI, can help with health-related queries, medication reminders, and appointment scheduling, significantly reducing nursing workload [3]. The time saved can then be used by nurses to provide individualized care to patients, personalized education and emotional support, tasks that are often neglected due to a lack of resources. Intelligent data processing and automation of administrative duties can enhance personalized patient care but also contribute significantly to nurses’ work–life balance [35]. Therefore, it seems that nursing professionals are eager to embrace the integration of AI into their everyday job duties, provided that their role is supplemented and not supplanted by algorithms [36].

Indeed, it is not easy to imagine that AI can provide for the patient in the way nurses do. Human touch, holism and empathetic communication are integral elements of nursing practice, all of which are out of AI’s scope of practice. These elements are also important for medical practice, but they are not as essential as they are in nursing. In many specialties, the physician’s role is mainly technical, and it is left up to nurses to be close to patients, communicate with them and implement medical decisions. Therefore, nurses may be justified in feeling more confident that AI is a tool that will upgrade their professional role without the risk of replacing them. Seen through the lens of the ethics of care, which regards caring relations as the moral core of nursing, this distinction matters. AI may support competence in carrying out a task, yet attentiveness to the unique person and responsiveness to their changing emotional and relational needs remain fundamentally human achievements [37]. This framework helps to explain why nurses tend to read AI as a support for care rather than a stand-in for it.

It needs to be noted, though, that we can never be certain of how the situation will evolve in the long run. Social, emotionally responsive robots are already used in nursing care, acting as supportive assistants to enhance elderly and chronic patient care by reducing loneliness, providing cognitive stimulation, and assisting with monitoring tasks [38]. These social robots are designed to respond to human interactions by engaging patients in conversation, playing games, and even providing affection [34]. The preliminary findings of a 2025 scoping review focusing on social robots with physical bodies in healthcare (and excluding surgical robots) suggest that this is an area of great interest worldwide; issues of trust and acceptability have been identified as main barriers, but these can be overcome as technology advances and social robots’ usefulness is continuously asserted [39]. Another issue that needs to be considered is the rise of artificial empathy, as many AI applications can discern emotional states based on speech analysis and facial recognition technologies, and respond accordingly by simulating empathic responses [40]. To be sure, affective AI systems lack authenticity [41], and research shows that patients prefer human over artificial empathy, even though they tend to rate AI empathy higher [42]. However, future developments could perfect this important aspect of nursing care in responsive AI technologies, thus adding to the uncertainty of predictions for the role transformation of nurses in the long run. Two developments deserve closer attention than they have received thus far. The first is the rise of relational and conversational agents built on large language models. Unlike scripted assistants, these can sustain open-ended dialogue, and they are already being explored as companions in chronic and elderly care settings [43]. Their fluency makes them more engaging, but also more persuasive, which raises the stakes of any error. The second is the dual use of the same affective technology. Systems that read a patient’s face and voice to infer emotion depend on models that can equally synthesize a convincing face and voice, so that a patient’s own likeness or speech can be fabricated and used to deceive carers, relatives, or the patient themselves [44]. Emotional AI in nursing therefore cannot be judged only by the comfort it provides. Its potential for impersonation and manipulation belongs in the same discussion.

### 3.3. The Changes in the Traditional Role of Patients

As previously implied, AI has the potential to radically transform the patient’s experience, leading to highly personalized approaches and improving outcomes at all levels. What matters for our discussion is the ways in which patients can become more active in their individualized care and increase their autonomy with the use of AI technologies.

In relation to physicians, we have already discussed that prompt prognosis and diagnosis can offer patients or prospective patients the opportunity to engage in the management of their health at an early phase, prior to the occurrence of physical deteriorations that leave them with fewer options. In addition, the time that physicians save as a result of AI’s automation can be used to provide more detailed information to patients, explore their preferences and mutually agree on tailor-made treatment plans, all of which increase patients’ autonomy within the therapeutic relationship [45]. The same applies to nurse–patient interaction, as nurses are also able to save considerable amounts of time thanks to AI’s handling of administrative duties, allowing them to devote more time to patients’ individualized care. But the most important changes in autonomy come from the opportunities presented to patients and their caregivers to use AI applications on their own. AI can be a valuable patient companion, offering personalized guidance on diet, physical exercise, and lifestyle choices. In addition, wearable devices and electronic health records processing can use individual health data to identify potential risks and suggest changes in treatment plans or prompt them to visit health professionals [30]. Apart from active self-management of their health, individuals can also use AI to enhance their health literacy and medical information-seeking abilities [42], further increasing their autonomy levels. AI-powered chatbots can offer personalized information and advice, allowing individuals to express their concerns and receive emotional support, even in geographically remote areas [46]. Therefore, it can be asserted that the traditional role of patients is empowered by AI, which is a key concern in modern healthcare.

Still, there are risks that cannot be neglected. Over-reliance on AI may hinder patients’ autonomy, especially if they are unable to discern accurate medical information from misleading data [45]. One could claim that the continuing progress of deep learning applications will eventually mitigate the problem of misinformation, but the way that inexperienced individuals interpret valid medical information can still lead to many misunderstandings, and experts warrant caution with regard to unsupervised deployment of AI virtual healthcare assistants for the use of the general public [47]. In addition, AI models may be biased, which in turn expands health disparities and threatens patients’ autonomy [48]. A final concern is that AI systems are vulnerable to adversarial attacks, where maliciously crafted inputs can manipulate their outputs [43]. Therefore, it is essential to strengthen systems’ defenses, reduce bias, and find appropriate ways of directly communicating with laypersons seeking medical advice. The extent to which these goals will be achieved is of crucial importance for a meaningful transformation of the patient role, which should be manipulation-free and with increased autonomy. The sharpest version of this problem is self-diagnosis and self-treatment. Increasing numbers of patients are turning to general-purpose chatbots to interpret symptoms and to decide whether and how to act, often before contacting a professional or instead of doing so. The accuracy of such advice is uneven, and studies of large language models used for self-diagnosis show that responses can be ambiguous or unsafe and are easily misread by people without clinical training. The answer is not to pretend that patients will stop. A more realistic response joins three things: public-facing AI tools that are explicit about their limits and that signpost when to seek care, basic health and digital literacy so that users can weigh what they read, and a clinical stance that treats a patient’s AI-derived beliefs as a starting point for conversation rather than something to dismiss. Handled this way, self-directed use of AI can widen autonomy instead of quietly eroding it.

## 4. Discussion

Based on all of the above, it is evident that the ongoing transformation of traditional roles in the therapeutic relationship can take different directions. It may be subject to technological advances that are yet to be seen, and appropriate measures and regulations need to be enforced. In what follows, we will discuss in more detail the main factors that influence, in positive and negative ways, the status of health professionals and patients in the new era of precision medicine and individualized care.

### 4.1. Outperformance Claims

The first issue that needs to be considered is the general assumption that AI systems can handle a variety of tasks more effectively than human individuals. Are these claims always supported by concrete evidence, or are there cases in which AI’s capabilities are overstated? If these systems have been tested in experimental settings and not in real clinical practice, their effectiveness remains uncertain. Research findings may be interpreted in overly positive ways, but without clarifying what AI can and cannot do in practice [16]. Therefore, before implementing digital solutions that have such a significant impact on the therapeutic relationship, outperformance claims should be specific and empirically grounded. Appropriate regulatory bodies need to evaluate AI’s safety and efficacy, and also provide guidance as to what kind of evidence is required by manufacturers and researchers for AI systems to be used as clinical companions [49]. It also helps to be precise about which kind of system is being judged. Traditional clinical decision support systems, including rule-based expert systems, are usually built and validated for a narrow, well-defined task, so their performance can be checked against a clear benchmark. Contemporary machine learning models are valued for the opposite reason, because they generalize to new and atypical cases; it is in these situations that their behavior is least predictable and hardest to verify. Therefore, humans cannot be fairly compared against AI in an abstract sense. It is the clinician working with a validated tool against the clinician working with a model whose outputs on unfamiliar inputs carry the very uncertainties that reviews of healthcare AI have repeatedly flagged [37]. Outperformance claims that skip this distinction tend to lend the new systems the reliability of the old ones [16].

### 4.2. Time Management

As noted earlier, AI can save a considerable amount of time for all healthcare professionals by processing medical data and producing results faster than any human individual, and also by automating administrative tasks and bureaucratic procedures. We presumed that this time can be used to enhance precision medicine, individualized care and patient autonomy, as physicians and nurses are able to interact more with patients and informal carers, understand their preferences, analyze their needs, offer information, and answer any queries. However, this may not be the case; the use of AI may introduce further time constraints for healthcare systems.

The first issue that needs to be considered is the one of explainability. Algorithms are complex, and the ways in which they reach conclusions may not be immediately clear. If healthcare professionals do not want to simply rely on AI without understanding its functioning and reasoning, they need to devote time and energy to and be properly trained in interpreting AI-powered results and suggestions [50]. This requirement also constitutes a shift in the traditional role of healthcare professionals; for instance, a new hybrid technical–medical curriculum has already been introduced in the Netherlands, leading to the creation of a “technical physician” degree [51]. Once healthcare professionals have understood AI’s functioning, they will need time to explain it to patients if they truly want to respect their autonomy. It is already difficult enough to provide simplified medical information to laypersons and secure their informed consent, and it is expected that the additional explanations of AI functioning will add to this difficulty, especially for patients with lower levels of digital literacy [52]. A further limitation is worth stating plainly. Explainability has a ceiling that no amount of training can remove, because many high-performing models are black boxes whose internal decision paths cannot be fully reconstructed, not even by their developers. Tracing how a given input moves through millions of parameters to a particular output is, in practice, out of reach, so what professionals obtain is usually a plausible account of a decision rather than its actual mechanism [52]. This has important implications for the therapeutic relationship because a clinician who cannot fully explain why a model recommends a given course of action faces a real limit on shared decision-making and informed consent [37].

The second issue to be considered is the time required by health professionals to clarify false beliefs that laypersons may develop if they use AI for medical information. For instance, a 2025 study found that GPT-4.0 responses are often ambiguous, and that its medical advice should be accepted with caution [53]. Physicians are already struggling with misinformation about diseases and treatments to which many internet users are susceptible, as they have access to various medical sources but often lack the means to properly interpret what they read. On the one hand, patients are empowered by AI tools that offer them medical advice, but, on the other, misunderstandings are common and medical experts must re-educate those who become victims of false health beliefs [54]. Therefore, although AI has the potential to radically enhance autonomy for both professionals and patients by allowing more time for human interaction, there is also the possibility of reducing it if lengthy and detailed explanations are in order.

### 4.3. Trust and Accountability Issues

In addition, the explainability of AI technology is directly related to the level of trust between health professionals and patients. Even when there is ample time for explanations and understanding, there will still be cases with highly complex deep learning models in which healthcare professionals may feel pressured to rely on decisions without fully understanding their rationale [55], and may be unable to fully inform patients about these decisions. This could seriously undermine trust in the therapeutic relationship by transforming health professionals into mere conveyors of AI’s will. Trust means reliance to someone who has knowledge and skills but also demonstrates good intentions and commitment to professional and humanitarian values; therefore, it cannot be placed on machines and algorithms. We can claim that an AI tool is reliable, but we cannot say that it is trustworthy [56]. As Savulescu et al. suggest, to trust AI is metaphorical language for trusting those who design and use it [15]. Therefore, in essence, patients have to place their trust in both healthcare professionals and AI programmers, although it is highly improbable that they will meet the latter in person. This mandatory trust in programmers threatens patients’ autonomy, as it reintroduces some form of paternalism into the therapeutic relationship. Algorithms guide therapeutic decisions based on values and priorities that they have been programmed to respect, but these values and priorities may not reflect individual patients’ values and priorities. It is thus important to ensure that health professionals will be able to keep a balance between patients’ unique preferences and AI programmers’ general directions. Trusting the designers of AI is necessary, but not sufficient. The people who build a model are not its only authors. What a system returns is shaped at least as much by the data assembled to train it, much of which is gathered and labeled far from any clinic and is of uncertain quality. High-quality clinical evidence is scarce and accrues slowly, so models often learn from what is abundant rather than from what is best, and they tend to reproduce statistically dominant patterns rather than the reasoning suited to the patient in front of the clinician [37]. Trusting AI therefore means trusting a long and largely invisible chain that runs from data collection through training to deployment, which makes transparency harder to secure than the language of trusting the programmers suggests [18].

This brings us to the issue of accountability, which is of utmost importance for the future role of healthcare professionals. What happens if an AI-aided therapeutic decision proves to be false or miscalculated and leads to patient harm? Can we hold AI programmers responsible for bad outcomes, and what would this mean for the therapeutic relationship? Legally speaking, these questions remain unanswered as the appropriate regulatory bodies need to address these accountability concerns and set clear guidelines, which should be harmonized to the greatest possible extent between different countries and jurisdictions [57]. Regulations and guidelines should also designate a minimum standard of care for AI-driven interventions so as to set grounds for negligence claims upon failure to meet this standard [58]. However, as previously discussed, the general idea is that algorithms should enhance and not replace human decision-making. Clinician oversight should be retained, and responsibility for final decisions should be shared appropriately between healthcare professionals and patients [59]. AI must be effectively managed so that health professionals do not learn to overly rely on it, and gradually waive their clinical, legal and ethical responsibility toward patients.

The goal to keep human practitioners at the forefront of accountability is particularly important when it comes to precision medicine and individualized care. While recent discussions regarding physician acceptance and the utility of clinical AI have shifted from direct replacement to implementation and integration [60], the adoption of AI continues to present the risk of transferring decision-making authority from humans to machines, especially when decisions involve complex data processing that hinders interpretability and explainability. Physicians and other healthcare professionals should serve not only as primary users of AI technologies, but also as active contributors to their development [61]. Since they possess unique insights into clinical practice, they can guide AI developers in designing tools that address genuine clinical needs. This active involvement will potentially diminish the risk of routinizing the use of AI and following its suggestions unreflectively, thus making it clear that human oversight is crucial for the protection of health professionals’ roles in the therapeutic relationship.

### 4.4. The Cost of AI

Finally, an important consideration regarding the ways in which AI transforms the healthcare industry and those involved within is how much it will cost. Economic predictions show “tremendous” cost savings as a result of using AI tools in diagnosis and treatment [62], especially if healthcare organizations choose to create their own AI tools instead of buying foundation models [63]. There is of course the issue of costly upgrades that will be needed in various healthcare areas in order for AI to be effectively used—for example, diagnostic images have to be of the greatest quality to meet the AI systems’ high standards, whereas unstable internet connection restricts their processing speed [12]; but let us suppose that, in the long run, upgrades are cost-effective due to cost reductions achieved by AI. The main question is the impact of these savings on health professionals’ practice. Will they see a significant rise in their share of income? Will they have more job opportunities? What if the cost reduction achieved by AI means that fewer health professionals are needed, or that some of their services should be cheaper due to AI’s fierce competition? What will the rise of AI mean for older health professionals who will not be able to adapt to this new reality?

There is global concern that AI will lead to considerable job losses, and the healthcare sector is no exception [64]. As we ascertained earlier, human oversight and accountability are essential, but this statement does not say anything about future numbers of health professionals needed or their remuneration figures. The perceived importance of their roles may be seriously diminished if physicians and nurses feel that healthcare organizations invest more in AI than in human employees, or that AI manufacturers gain significant profits while health workers’ jobs and salaries can be sacrificed for greater cost-effectiveness. This professional role devaluation can have detrimental effects on the attractiveness of healthcare positions, leading the brightest students to pursue different career pathways, and changing the societal acceptance of health workers. Professional organizations must ensure that cost reductions achieved by the implementation of AI will not backfire to physicians, nurses and members of other related disciplines, and policymakers must observe managerial practices of health organizations to safeguard respect and high status for health professions.

## 5. Strengths, Limitations and Future Directions

Strengths: This review brings together evidence that is usually considered separately, across medicine, nursing and the patient perspective, and reads it through a single lens: the redistribution of roles within the therapeutic relationship. By organizing the analysis around four cross-cutting factors and an explicit ethics-of-care framework, it offers a structure that others can apply to particular specialties or settings.

Limitations: Two limits should be stated plainly. First, this is a narrative review, so the search was purposive rather than exhaustive, and a different selection of sources could shift the emphasis. Second, and more important, the evidence base itself is uneven. Much of the literature on AI and role transformation is conceptual, or draws on early-stage studies, pilots and experimental settings, while high-quality real-world data on outcomes, costs and the durability of role changes is still scarce. Several claims in this article therefore rest more on theoretical analysis than on robust empirical findings, and we have tried to mark this where it matters. The conclusions should be read with that balance in mind.

Future directions: The most useful next step is empirical. The field needs prospective and longitudinal studies exploring how roles actually change when AI is deployed, and not only how a tool performs in isolation, alongside real-world data on cost and on patient and professional outcomes. Work on relational and conversational agents, on the safe management of patient self-diagnosis, and on clear lines of accountability for AI-assisted decisions would help move the debate from principle to practice.

## 6. Conclusions

As the latest evidence clearly indicates, AI technologies are currently augmenting the scope of practice for precision and personalized medicine in unprecedented ways. The transformation that AI inflicts on the traditional roles within the therapeutic relationship is also an undeniable fact. Given AI’s massive potential to improve patient outcomes and reduce healthcare costs, resistance to this change looks like denial of reality. What is less certain, however, is the direction that this transformation will take in the future, as it depends on various factors. Many scholars have expressed concerns and made proposals about the appropriate use and regulation of AI applications in healthcare, which will ensure that the transformation will take place in a meaningful way for both health professionals and patients, increasing their autonomy and safeguarding the social value of their roles. Drawing on the four themes examined above, the main recommendations can be summarized as follows:

- Outperformance: require that claims of superior performance be demonstrated in real clinical practice and on the case mix a service actually treats, not only in curated experiments, before a tool is allowed to reshape a role.
- Time: protect the time that AI frees so that it returns to patients, since the gain is lost if it is reabsorbed by oversight, training and the work of explaining AI to patients.
- Trust and accountability: keep the responsibility with the humans who commission and use AI, set a minimum standard of care for AI-assisted decisions, and tell patients clearly when AI informs their care.
- Cost: measure any savings attributed to AI against full implementation costs and do not realize them by cutting clinical posts or pay so that the social value of the professions is safeguarded.
- Across the four themes: AI should complement professional skills rather than replace them, and unreflective over-reliance should be avoided by professionals and patients alike.
- Above all: communication with AI lacks genuine empathy, so the relational core of care must remain a human responsibility, and AI-assisted decisions must stay explainable enough to support shared decision-making.

Above all, what needs to be kept in mind is that AI is not just a tool, as is so often stated. It promotes breakthrough innovation at such a rapid pace that it tempts us to use it without critical reflection. Human intelligence can be seen as inferior to AI—yet this is a mistake, as they are two different kinds of intelligence. AI’s greatest strength lies in the automation of complex procedures. Human intelligence excels in areas requiring empathy, judgment, and original, context-driven thought. The increasing use of AI tools should not blind us to this fact. The real danger might be related to humans being willing to handle over control to machines, limiting themselves to the role of passive observers. The exact way of proceeding in the future with the use of AI remains to be seen, both in the healthcare industry and all the other areas of its influence. However, the wealth of ethical concerns, warnings and suggestions on its proper use, expressed by prominent scientists and bioethicists in numerous papers, interviews and journal articles, leaves room for optimism. At the very least, healthcare scholars appear ethically aware and vigilant in the face of the ongoing role transformations that are taking place.

Returning to the questions set out at the start, the role analysis shows that AI redistributes rather than abolishes the work of physicians, nurses and patients, moving professionals toward oversight and interpretation while giving patients new means of self-management. Whether this redistribution strengthens or weakens autonomy hinges on the four factors examined here: how credibly outperformance is demonstrated, how the time freed by AI is actually spent, how trust and accountability are anchored in people rather than in tools, and how cost pressures are managed so that they do not quietly decide clinical questions. Read through the ethics of care, the measures that protect the therapeutic relationship are the ones that keep attentiveness and responsiveness in human hands while letting AI take on the work it does well. The transformation is not something to resist or to welcome wholesale, but something to govern.

## Figures and Tables

**Table 1:** Summary of AI-driven role shifts in the therapeutic relationship.

Role	Direction of Role Shift	Main Opportunity	Main Risk	Human Anchor (Safeguard)
Physicians	From doing toward overseeing and interpreting	Faster, more accurate prognosis, diagnosis and treatment planning	Reduction to a validating role; over-trust in opaque models	Clinical judgment on atypical cases; final responsibility for decisions
Nurses	Augmentation rather than substitution	Time and tools returned to direct, individualized care	Affective AI and social robots displacing the relational core of care	Attentiveness and responsiveness as human achievements (ethics of care)
Patients	From passive recipient toward active self-manager	Greater health literacy, monitoring and autonomy	Misinformation, biased advice, unsafe self-diagnosis and self-treatment	Clinician guidance, signposting, and health and digital literacy

## Data Availability

No new data were created or analyzed in this study. Data sharing is not applicable to this article.

## Abbreviations

The following abbreviations are used in this manuscript:

AI	Artificial Intelligence
GPT	Generative Pre-trained Transformer
